# Primary Benign Phyllodes Tumour of the Labia Minora: An Uncommon Entity

**DOI:** 10.7759/cureus.31616

**Published:** 2022-11-17

**Authors:** Harrypal Panesar, Harjit Dhaliwal

**Affiliations:** 1 Otolaryngology, University Hospitals of North Midlands NHS Trust, Stoke-on-Trent, GBR; 2 Obstetrics and Gynaecology, Royal Bournemouth Hospital, Bournemouth, GBR

**Keywords:** benign tumours, vulva, gynaecological surgery, labia minora, phyllodes tumour

## Abstract

A phyllodes tumour of the vulva is a rare, well-illustrated benign neoplasm having characteristic histo-morphological features similar to a phyllodes tumour of the breast. We report a case of a primary benign phyllodes tumour in a 28-year-old female patient. She was seen in an outpatient clinic presenting with a slow-growing, non-painful lesion on her vulva. Examination revealed a 2 cm cyst located on the labia minora. Complete excision of the cyst was achieved, and pathological examination revealed a benign phyllodes tumour of the vulva. Hallmarks of this rare pathology are classically a leaf-like architectural configuration and fronds projecting into the cystic spaces on a low-power magnification. To date, a grading or classification of these vulva tumours has not been established due to their rarity. There have been very few reported cases of phyllodes tumours occurring on the vulva and fewer still affecting the labia minora. Continued surveillance for recurrence should be adopted.

## Introduction

Phyllodes tumours (PTs) of the vulva are exceedingly rare tumours that are mostly benign. There have only been 18 cases reported so far in the literature [1]. They usually present as a unilateral, painless mass arising most commonly in the labia majora. Other locations include the interlabial sulcus, mons pubis and periclitorial sites. There has only previously been one reported case affecting the labia minora. The differential diagnosis includes other biphasic tumours such as fibroadenoma, papillary hidradenoma and chondroid syringoma and extension of Mullerian adenosarcoma of the cervix into the vulva [2-5]. PTs are more commonly found within the breast and account for less than 1% of all breast neoplasms [6]. The majority are benign with a local recurrence risk of 17% [7]. In contrast, malignant PTs have a 27% risk of a local recurrence and a 22% risk of distant metastasis [7]. PTs of the vulva are histologically similar to phyllodes of the breast. The current mainstay of therapy is surgical resection with clear margins and continuous surveillance for recurrence is a must.

## Case presentation

A 28-year-old woman with no significant past medical history was referred for an evaluation of a non-infected, non-painful vulval lump that has been steadily growing over the years. On examination, there was a left labial cyst, which was 2x2 cm, located on the labia minora. It was mobile, non-tender and not adhered to other underlying structures. There was no evidence of inguinal lymphadenopathy. Complete excision of the cyst was performed.

**Figure 1:**
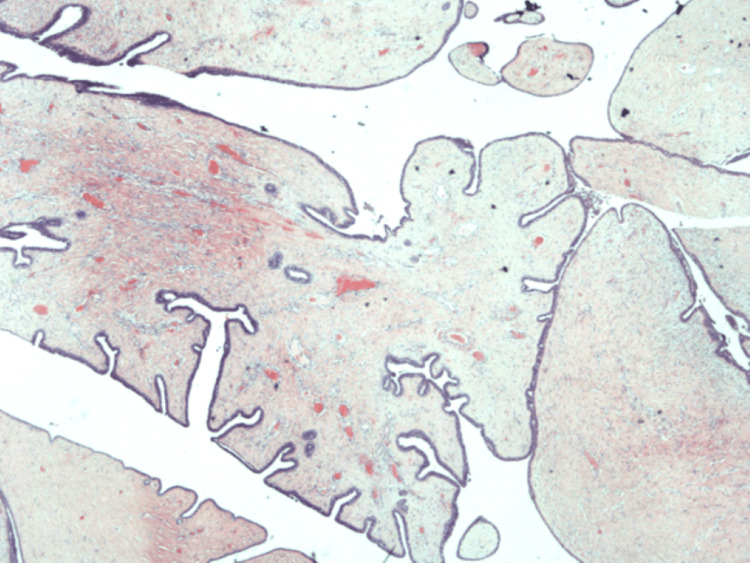
Microscopic examination showing a classic leaf-like configuration projecting into cystic spaces (H&E, x20)

**Figure 2:**
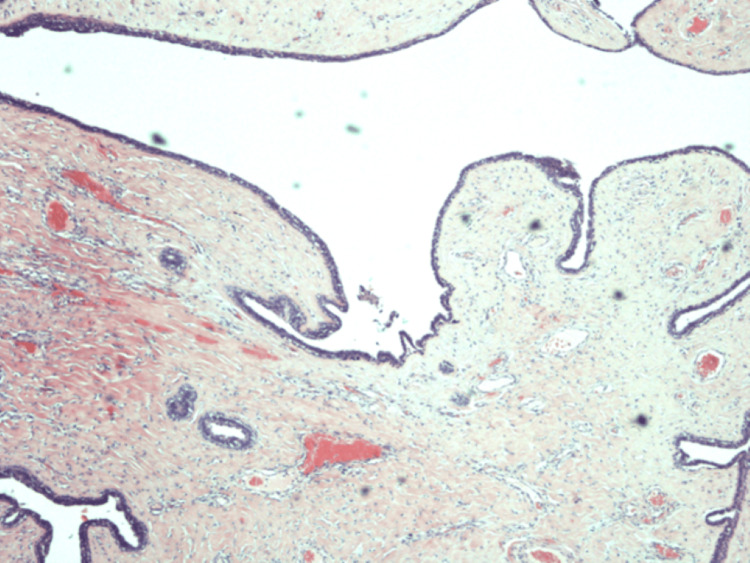
Microscopic examination showing the frond growths covered with a bilayer epithelium-myoepithelial layer with a secretory-type outermost layer (H&E, x40)

**Figure 3:**
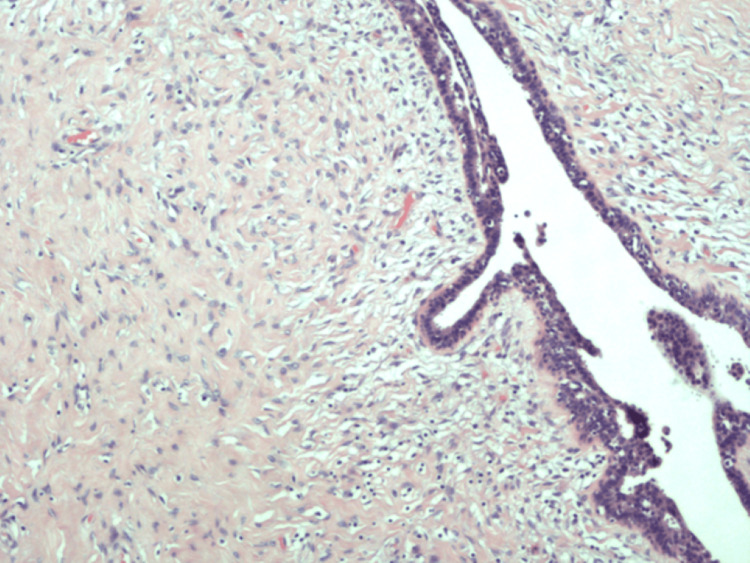
Microscopic examination of the lesion displaying stromal hypertrophy (H&E, x100)

Macroscopic examination revealed a 2 cm partly cystic greyish-white lesion. Areas of haemorrhage and necrosis were absent. The examination revealed classic leaf-like projections into cystic spaces (Figure 1), frond growths with an epithelium-myoepithelium bilayer (Figure 2) and stromal hypertrophy (Figure 3). The microscopic examination did not reveal any ectopic breast tissue or mammary-like glands around the lesion. There was no cellular atypia or mitosis seen in the stromal component. An immunohistochemical examination was not carried out. Hence, the diagnosis of benign phyllodes tumour of the vulva was made by routine histopathology examination.

## Discussion

Phyllodes tumours of the vulva are uncommon benign tumours. They have a similar histomorphologic biphasic, leaf-like configuration pattern to its counterpart in the breast. They were first reported by Hartung et al. in 1872, who were the first to report mammary-like glands in the vulva [2,8].

Phyllodes of the vulva usually present as a solitary unilateral, well-circumscribed and painless mass. The labia majora, minora and interlabial cleft are the most frequent sites of presentation. Other less frequent sites include the mons pubis, periclitoral and para median area of the perineum and around the anus [8,9]. There also has been a case series of phyllodes tumours involving the prostrate and the seminal vesicles; hence, the presence of specific breast tissue is not a prerequisite for their development, as these tumours were in regions away from the milk line ridges. Immunohistochemistry stains were negative for oestrogen but showed a positive reaction to prostate-specific antigen and prostatic acid phosphatase [10].

There is a wide variation in the age group, ranging from 17-69 years and commonly manifests in the third to fourth decade of life in women. The tumour size varies from <1 cm up to 6.6 cm with a variable growth rate. In our case report, the tumour was first noticed two years ago and had been slowly growing up to a size of 2 cm. Macroscopically, PTs are usually dirty-white, homogenous, well-delineated lesions with cystic or solid-cut surfaces. Microscopically, frond-like papillary projections or a polypoid appearance with a pushing margin are also characteristic features [11].

There are two theories that evolve around the pathogenesis of phyllodes of the vulva. The first theory stated these lesions originated from the presence of ectopic breast tissue representing caudal remnants of the milk line ridges [8]. However, a more recent and widely accepted theory argues that specific mammary-like glands (MLG), present in the anogenital region are attributed to the development of these lesions [12]. Evidence from the literature review does suggest that the concept of vulva ectopic breast tissue derived from mammary ridges is no longer acceptable and MLG being a normal constituent of the anogenital region being derived from cloacae rather than the mammary line ridges. These MLG mimic the mammary glands but are still distinguishable from eccrine and apocrine glands [12]. Van Der Putte refuted that these MLG arose from the ectopic breast tissue due to the following points: a) MLG have a much simpler configuration and a different acinar epithelium, b) a higher number of MLG are present in the anogenital region compared to remnants of mammary ridges and its relationship is towards cloaca-derived tissues, and c) finally, a direct relationship to eccrine glands in the anogenital region [8,12]. Giger et al. also demonstrated a strong NY-BR-1 expression of PT of the vulva with a similar strong expression from nearby mammary-like anogenital glands, suggesting that these tumours in the vulva arise from MLG [13]. MLG often exhibit oestrogen receptor and progesterone receptor expression rather than ectopic breast tissue. Similar to the mammary glands, these glands have the capacity to branch into lobuli and form acini [11]. In this case, histology revealed no evidence of ectopic breast tissue.

The differential diagnosis of phyllodes tumour of the vulva includes other biphasic tumours such as fibroadenoma, chondroid syringomas, papillary hidradenomas, and Mullerian adenosarcoma of the cervix that has metastasised in the vulva may have similar leaf-like architecture and biphasic pattern with increased mitotic activity, greater stromal cellularity, and increased periglandular stromal condensation [14,15]. Chondroid syringoma may resemble phyllodes because of its biphasic nature, but the absence of the leaf-like structure and the presence of cartilaginous components within the myxoid stroma of the syringoma helps in the differentiation between the two entities. Fibroadenomas though rare are more common than PT [16]. They mainly lack the characteristic leaf-like pattern as seen in PT. The assessment of stromal cellularity is important as fibroadenomas are less cellular without atypia whilst PT displays increased cellularity [15]. Papillary hidradenoma may be grossly similar to PT due to its cystic nature but lacks the stromal component. In addition, the papillary structures are surrounded by a double epithelial layer and have focal apocrine protrusions.

Immunohistochemistry is also useful and aids in differential diagnosis. Secretory epithelial cells are positively stained with oestrogen and progesterone and the myoepithelial cells are stained with p63, SMA and S100 [14]. The stroma is negatively stained with oestrogen, progesterone, WT1 and CD10, and positive staining is seen with CD34, Vimentin and SMA [14]. The Ki-67 proliferation rate was seen in 1-15% of the cases [11].

## Conclusions

We report an exceedingly rare benign phyllodes tumour of the vulva. They usually present as painless, slow-growing and unilateral masses. Due to rarity and paucity in the literature, a histopathological grading has not been established. Local recurrence is a possibility if there is an incomplete excision of the tumour. Follow-up is warranted in all patients with this tumour as the prognosis is unclear.

## References

[1] Fujii DT, Korzen CA, Levine TC, Heitmann RJ (2019). Phyllodes tumour of the labia minora. BMJ Case Rep.

[2] Mannan AA, Kahvic M, Aziz AH (2010). Phyllodes tumor of the vulva: report of a rare case and review of the literature. Am J Dermatopathol.

[3] Chulia MT, Paya A, Niveiro M, Ceballos S, Aranda FI (2001). Phyllodes tumor in ectopic breast tissue of the vulva. Int J Surg Pathol.

[4] Kazakov DV, Spagnolo DV, Kacerovska D, Michal M (2011). Lesions of anogenital mammary-like glands. An update. Adv Anat Pathol.

[5] Kazakov DV, Spagnolo DV, Stewart CJ (2010). Fibroadenoma and phyllodes tumors of anogenital mammary-like glands: a series of 13 neoplasms in 12 cases, including mammary-type juvenile fibroadenoma, fibroadenoma with lactation changes, and neurofibromatosis-associated pseudoangiomatous stromal hyperplasia with multinucleated giant cells. Am J Surg Pathol.

[6] Zhang Y, Kleer CG (2016). Phyllodes tumor of the breast: histopathologic features, differential diagnosis, and molecular/genetic updates. Arch Pathol Lab Med.

[7] Tan PH, Thike AA, Tan WJ (2012). Predicting clinical behaviour of breast phyllodes tumours: a nomogram based on histological criteria and surgical margins. J Clin Pathol.

[8] van der Putte SC (1994). Mammary-like glands of the vulva and their disorders. Int J Gynecol Pathol.

[9] Denlinger LN, Lokhandwala PM, Abendroth CS (2015). Benign phyllodes tumor of the vulva: a case report and literature review. Rare Tumors.

[10] Bostwick DG, Hossain D, Qian J (2004). Phyllodes tumor of the prostate: long-term followup study of 23 cases. J Urol.

[11] Lee S, Nodit L (2014). Phyllodes tumor of vulva: a brief diagnostic review. Arch Pathol Lab Med.

[12] Van der Putte SCJ (1991). Anogenital “sweat” glands. Histology and pathology of a gland that may mimic mammary glands. Am J Dermatopathol.

[13] Giger OT, Lacoste E, Honegger C, Padberg B, Moch H, Varga Z (2007). Expression of the breast differentiation antigen NY-BR-1 in a phyllodes tumor of the vulva. Virchows Arch.

[14] Heffernan TP, Sarode VR, Hoffman B, Lea J (2010). Recurrent phyllodes tumor of the vulva: a case report with review of diagnostic criteria and differential diagnosis. Int J Gynecol Pathol.

[15] García-Rostán y P erez GM, Troyas RG, Bercero EA (1995). Müllerian adenosarcoma of the cervix: differential diagnosis, histogenesis and review of the literature. Pathol Int.

[16] Baisre A, Heller DS, Lee J, Zheng P (2002). Fibroadenoma of the vulva. A report of two cases. J Reprod Med.

